# Genome sequence of *Mycobacterium abscessus* phage P3MA

**DOI:** 10.1128/mra.00169-25

**Published:** 2025-05-22

**Authors:** Antonio Broncano-Lavado, Françoise Roquet-Banères, Laurent Kremer, Daniel A. Russell, Deborah Jacobs-Sera, Thomas G. Reidy, John J. Aguilera-Correa, Jaime Esteban, Graham F. Hatfull, Meritxell García-Quintanilla

**Affiliations:** 1 Department of Clinical Microbiology, Instituto de Investigación Sanitaria Fundación Jiménez Díaz (IIS FJD), Madrid, Spain; 2 CIBERINFEC-CIBER de Enfermedades Infecciosas, Madrid, Spain; 3 Centre National de la Recherche Scientifique UMR 9004, Institut de Recherche en Infectiologie de Montpellier (IRIM), Université de Montpellier, Montpellier, France; 4 INSERM, IRIM 131821 , Montpellier, France; 5 Department of Biological Sciences, University of Pittsburgh 6614, Pittsburgh, Pennsylvania, USA; Loyola University Chicago, Chicago, Illinois, USA

**Keywords:** bacteriophage genetics, bacteriophage therapy, *Mycobacterium abscessus*

## Abstract

Mycobacteriophage P3MA is a newly isolated bacteriophage recovered from the Manzanares River in Madrid, Spain, using *Mycobacterium abscessus* 330 as a host strain. P3MA has a 41,151 bp genome with 63 predicted protein-coding genes and is closely related to prophages identified in several *M. abscessus* genomes grouped in Cluster HB.

## ANNOUNCEMENT

*Mycobacterium abscessus* is ubiquitous in the environment (1) and can cause pulmonary and disseminated infections in persons with cystic fibrosis or various immune disorders (2, 3). Antibiotic treatment of these infections is challenging, with many strains having intrinsic and acquired antibiotic resistance (4, 5). Many mycobacteriophages have been isolated on the non-pathogenic *Mycobacterium smegmatis* (6), a few of which infect some *M. abscessus* strains and have been used therapeutically (7). Over 2,500 mycobacteriophages have been sequenced and are grouped in clusters, subclusters, and singletons according to their genomic relationships (6).

Approximately 75% of *M. abscessus* strains carry at least one integrated prophage, and some carry six or more (8). These prophages can be similarly grouped into clusters/subclusters/singletons (MabA, MabB, etc.), and these have been consolidated into a unified system with the *M. smegmatis* phages (8). A high proportion of these prophages bioinformatically appear to be functional, and some are capable of lytic propagation on susceptible hosts, although their host range is narrow among *M. abscessus* clinical isolates (9–11). Surprisingly, relatively few phages have been isolated on *M. abscessus* strains directly, given the likely environmental prevalence of the spontaneously induced prophages.

Phage P3MA was isolated using the clinical isolate *M. abscessus* strain 330 and water from the Manzanares River in Madrid. The water was filter sterilized and incubated with *M. abscessus* 330 and cations for 3 days at 37°C in tryptic soy broth (TSB) medium. The phage was plaque-purified as described previously (12), and genomic DNA was isolated by extraction with phenol:chloroform:isoamyl alcohol (13). The genome was sequenced first using an Accel-NGS 1S Plus DNA Library kit on an Illumina MiSeq—yielding ~144,000 paired-end 250 bp reads—and then using an Illumina DNA Prep kit on an Illumina NovaSeq—yielding ~1.55 million paired-end 150 bp reads. Reads from both runs were trimmed using fastp (-q 30, -e 30, and -l 50) and assembled with Unicycler (14) version 0.5.1 and standard parameters, yielding a major contig of 41,151 bp with 63.4% G + C and an average genome coverage of 1,542-fold. Genome completeness and phage genomic termini were determined using Consed version 29 as previously described (15). A second contig of ~52.3 kbp assembled from 3% of the total reads, and PCR analyses confirmed that this contig likely corresponds to spontaneously induced particles of a prophage resident in strain *M. abscessus* 330.

**Fig 1:**
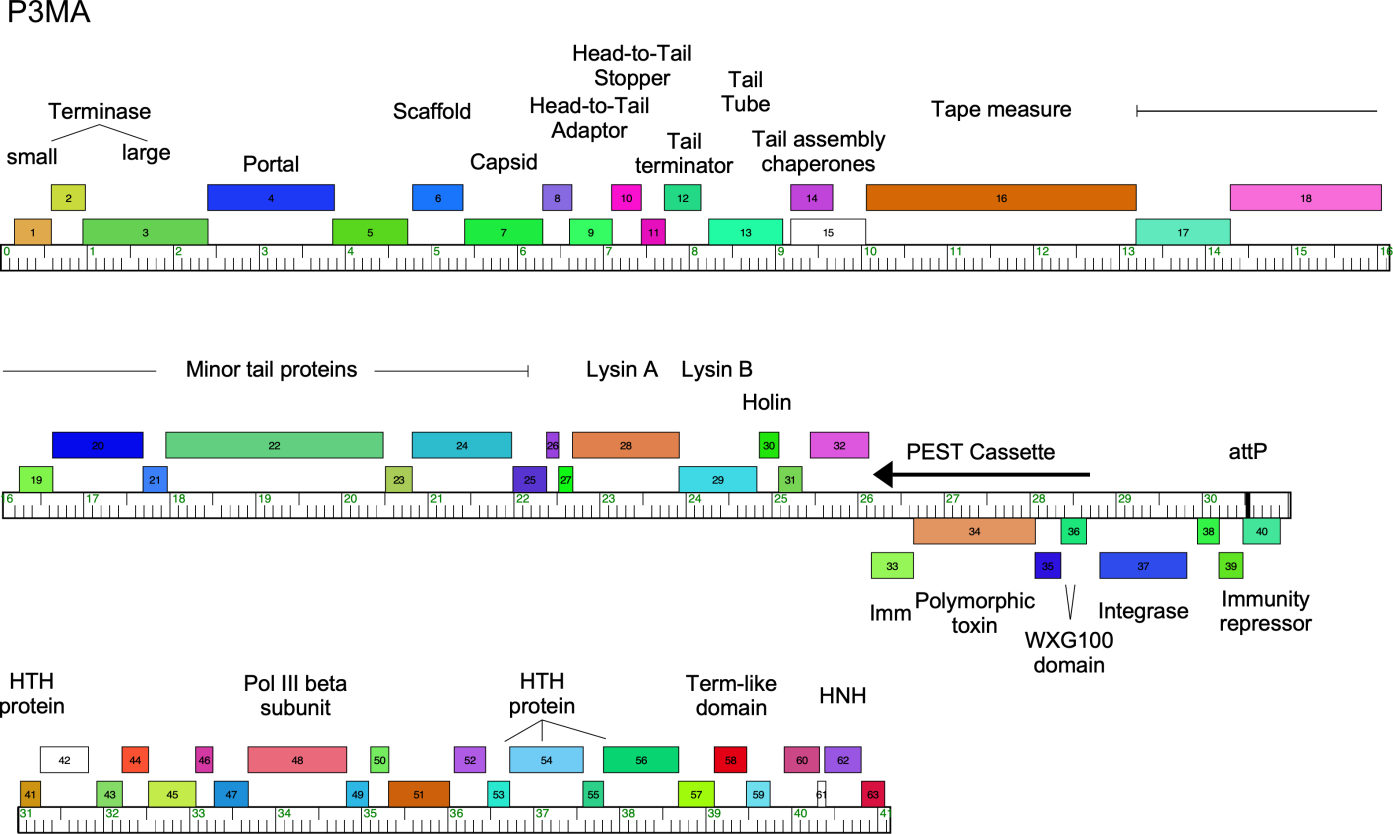
Genome organization of *Mycobacterium* phage P3MA. The genome is shown as a ruler with genes as boxes above (forward orientation) or below (reverse orientation). Genes are colored by family designations according to Phamerator Database Actino_Mab_Draft (version 32). Putative gene functions are indicated, including the predicted PEST cassette (8).

Bioinformatic analyses using GeneMark v.2.5p (16), Glimmer v.3.02 (17), Phamerator v32 (18), and DNA Master (http://cobamide2.bio.pitt.edu) predict 63 protein-coding genes and no tRNA genes (Fig. 1) (19). Unless otherwise noted, default parameters were used for all software tools. Putative gene functions were assigned to 36 (57%) genes, including a phage-encoded ESX-secreted toxin (PEST) system (8). Genome comparisons using BLAST (20) showed that P3MA is nearly identical to a previously described *M. abscessus* prophage, prophiT50-1 (21), grouped in Cluster HB (MabB); the two genomes differ by three single-nucleotide polymorphisms (SNPs), at coordinates 1,384, 8,917, and 30,945. The latter SNP is in an intergenic region and could influence gene expression; we note that P3MA forms clear plaques on *M. abscessus* 330.

To our knowledge, P3MA is the first spontaneously induced prophage isolated from an environmental sample. Its therapeutic potential would require engineering to be strictly lytic and likely removal of the polymorphic toxin; its host range among *M. abscessus* strains is unknown. Nonetheless, it suggests that other naturally occurring *M. abscessus* phages could be isolated from environmental samples.

## Data Availability

P3MA is available at GenBank with Accession No. PV089522. Sequencing reads are parts of the sequence read archive accession numbers SRX27671432 and SRX28386587.
